# *Edwardsiella tarda* Endocarditis Confirmed by Indium-111 White Blood Cell Scan: An Unusual Pathogen and Diagnostic Modality

**DOI:** 10.1155/2016/1082160

**Published:** 2016-01-17

**Authors:** Kayleigh M. Litton, Bret A. Rogers

**Affiliations:** Division of Cardiovascular Disease, Department of Medicine, University of Tennessee Medical Center Knoxville, Graduate School of Medicine, 1924 Alcoa Hwy, Knoxville, TN 37920, USA

## Abstract

*Edwardsiella tarda* is a freshwater marine member of the family Enterobacteriaceae which often colonizes fish, lizards, snakes, and turtles but is an infrequent human pathogen. Indium-111- (^111^In-) labeled white blood cell (WBC) scintigraphy is an imaging modality which has a wide range of reported sensitivity and specificity (from 60 to 100% and from 68 to 92%, resp.) for diagnosing acute and chronic infection. We describe a case of suspected* E. tarda* prosthetic aortic valve and mitral valve endocarditis with probable vegetations and new mitral regurgitation on transthoracic and transesophageal echocardiograms which was supported with the use of ^111^In-labeled WBC scintigraphy.

## 1. Case Presentation

A 68-year-old Caucasian male with prior mechanical prosthetic aortic valve implantation in 1995, mitral valve annuloplasty in 2010, and rate-controlled atrial fibrillation presented to an outside facility with a chief complaint of fatigue and body aches. Upon admission, he was febrile (38.2°C) and had a normal white blood cell count with a significant left shift (27% bands) as well as an elevated procalcitonin (38.63 ng/mL). He was previously very active, exercising for three hours, three to four times weekly; however, he had been feeling ill with fatigue and body aches for two days and spent the day prior to presentation lying on a hard ice pack, developing a sacral decubitus ulcer. Initial workup and management of his sepsis focused on the ulcer as a source. Therefore, blood cultures were drawn and he was started on broad-spectrum antibiotics, including vancomycin, piperacillin/tazobactam, and ciprofloxacin.

On day two of his hospital stay, 2/2 blood cultures drawn 10 hours apart grew* Edwardsiella tarda* which was sensitive to all antibiotics tested allowing refinement of the antibiotic regimen to ceftriaxone and levofloxacin. The patient was questioned about possible freshwater marine exposure, and he admitted to having a deep puncture wound in his hand with a used fish hook about two weeks prior to presentation.

The following five hospital days were complicated by a waxing and waning encephalopathy despite defervescence, sterile follow-up blood cultures, and normalization of the left shift on complete blood count differential. On hospital day number 7, his atrial fibrillation was poorly rate controlled so cardiology was consulted. A transthoracic echocardiogram was performed, which showed an echodensity on the mechanical aortic valve, concerning for vegetation. With this finding, his family requested his transfer to our facility.

**Figure 1:**
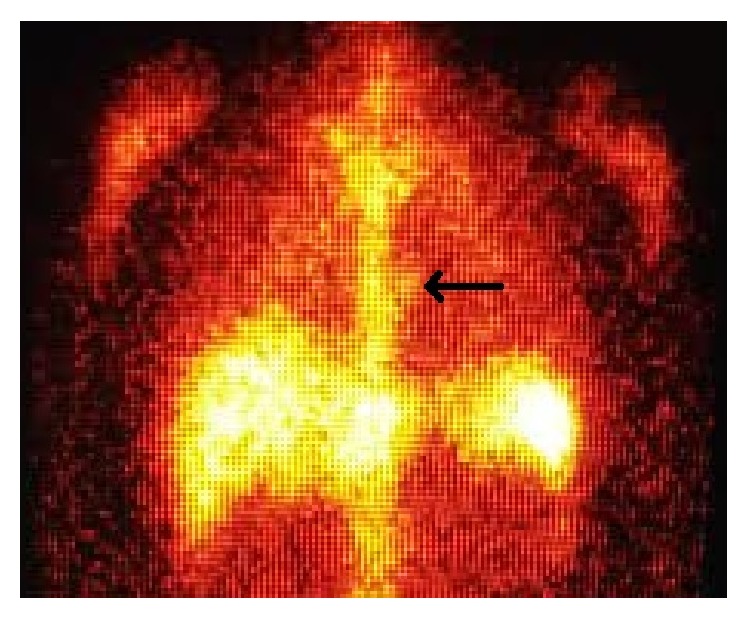
Indium-111 tagged WBC scintigraphy with focal area of asymmetric radiotracer uptake in the area of the mitral valve (arrow), coronal plane.

Upon arrival to our facility, levofloxacin and ceftriaxone were continued and a transesophageal echocardiogram was performed. The prosthetic aortic valve appeared to be well seated. The aortic valve leaflets were poorly visualized, but the previously repaired mitral valve demonstrated new moderate regurgitation and a vegetation on the anterior leaflet. With this rather confusing clinical picture of possible multivalve endocarditis, infectious disease and cardiothoracic surgery were consulted. Ultimately, upon discussion with the patient and consultants, the decision was made to obtain more information with an Indium-111- (^111^In-) labeled white blood cell (WBC) scan. This scan was chosen due to availability at our facility at the time. The ^111^In-labeled WBC scan showed a focal collection of radiotracer labeled white blood cells overlying the expected region of the mitral valve, consistent with endocarditis (Figure 1) as well as focal uptake over the sacrum, in the area of the decubitus. The patient was not a good candidate for valve surgery as it would be his third sternotomy; therefore, he was sent home after a total of 21 days in the hospital on indefinite suppressive therapy with levofloxacin. At 6-month follow-up, he has continued this regimen with no further complications and has gotten back to his active lifestyle.

## 2. Discussion

*Edwardsiella tarda* is a gram negative, motile, facultatively anaerobic rod which is most commonly isolated from freshwater marine organisms. It is not unheard of as a human pathogen, but it rarely causes extraintestinal infection [1, 2]. A review of the literature reveals one case report of successfully treated* E. tarda* prosthetic aortic valve endocarditis in a patient with AIDS who had sustained a catfish barb injury two weeks before the onset of symptoms [3]. Other cases have described extraintestinal manifestations including soft tissue infection, neonatal sepsis and meningitis, osteomyelitis, and vascular prosthesis infection [4–7]. Our patient likely developed* E. tarda* bacteremia after puncturing his hand with a used fish hook two weeks prior to presentation. The resultant fever, chills, and body aches caused him to lay for a sustained time on a hard ice pack, thus creating the sacral decubitus ulcer which was initially thought to be the source of infection.

The patient's cardiac anatomy made transthoracic and transesophageal echocardiogram challenging; however, he did meet one major and three minor Duke criteria. To meet major criteria, he had a possible vegetation on the prosthetic aortic valve as well as a vegetation on the mitral valve with a new mitral valve regurgitant murmur compared to his postoperative echocardiogram. To meet minor criteria, he had a fever at admission, a prosthetic heart valve, and positive blood cultures not meeting the definition of major criteria [8]. With one major and three minor criteria, we felt comfortable diagnosing endocarditis; however, the patient, a highly educated businessman, wanted further evidence before he was willing to accept lifelong suppressive antibiotics.

^111^In-labeled WBC scintigraphy for evaluation of endocarditis receives a rating of 3 for the American College of Radiology Appropriateness Criteria, which means it is rarely appropriate. The guidelines also state that there are situations where this imaging modality can be useful in isolated patients [9]. Other modalities such as 18F-FDG PET/CT and 99 m Technetium-HMPAO-labeled leukocyte SPECT/CT have also been shown as effective imaging options for prosthetic valve endocarditis, but both were unavailable at our facility at the time [10, 11]. Our patient's anatomy provided poor windows for echocardiographic examination and although he met Duke criteria to diagnose endocarditis the patient was unwilling to accept lifelong suppressive antibiotics without further information. Because of this, additional imaging obtained with ^111^In-labeled WBC scintigraphy was indispensable for helping the patient understand and accept his treatment. Although retrospective case reviews have suggested that ^111^In-labeled WBC scans may be useful for patient care less than half the time they are ordered, when ordered in a targeted manner, such as this case, they can be very valuable [12].
